# Genomic Diversity in Pig (*Sus scrofa*) and its Comparison with Human and other Livestock

**DOI:** 10.2174/138920211795564386

**Published:** 2011-04

**Authors:** Chunyan Zhang, Graham Plastow

**Affiliations:** 1400 College Plaza, Department of Agricultural, Food and Nutritional Science, University of Alberta, Edmonton, AB T6G 2C8, Canada

**Keywords:** Pig, genomic diversity, linkage disequilibrium, human, livestock.

## Abstract

We have reviewed the current pig (*Sus scrofa*) genomic diversity within and between sites and compared them with human and other livestock. The current Porcine 60K single nucleotide polymorphism (SNP) panel has an average SNP distance in a range of 30 - 40 kb. Most of genetic variation was distributed within populations, and only a small proportion of them existed between populations. The average heterozygosity was lower in pig than in human and other livestock. Genetic inbreeding coefficient (*F_IS_*), population differentiation (*F_ST_*), and *Nei*’s genetic distance between populations were much larger in pig than in human and other livestock. Higher average genetic distance existed between European and Asian populations than between European or between Asian populations. Asian breeds harboured much larger variability and higher average heterozygosity than European breeds. The samples of wild boar that have been analyzed displayed more extensive genetic variation than domestic breeds. The average linkage disequilibrium (LD) in improved pig breeds extended to 1 - 3 cM, much larger than that in human (~ 30 kb) and cattle (~ 100 kb), but smaller than that in sheep (~ 10 cM). European breeds showed greater LD that decayed more slowly than Asian breeds. We briefly discuss some processes for maintaining genomic diversity in pig, including migration, introgression, selection, and drift. We conclude that, due to the long time of domestication, the pig possesses lower heterozygosity, higher *F_IS_*, and larger LD compared with human and cattle. This implies that a smaller effective population size and less informative markers are needed in pig for genome wide association studies.

## 1. INTRODUCTION

Worldwide, domestic pigs can be divided into two main clades: Asian- and European-types, which diverged from each other around 58,000 years ago. The European wild boars were domesticated at least in the 4th millennium BC and then rapidly spread throughout Europe [1, 2]. Asian pigs were thought to have been introduced into Europe during the late 18th and early 19th centuries [3], and recent studies suggest this has had a significant impact on the diversity of these breeds. Significant breed development took place from the 18th Century, followed by extensive organized genetic improvement (artificial selection) through the application of quantitative genetics theory in the second half of the 20^th^ Century. Even so a large number of local breeds are maintained especially in China. It is commonly held that current European and Asian pig breeds are domesticated from different ancestors that might have different genome wide diversity and extent of linkage disequilibrium (LD). 

Initial genetic studies in the pig mainly focused on investigating the genetic diversity of a breed at relatively small numbers of individual sites, which is essential for sustainable management of genetic resources for future utility. With the identification of the first useful DNA marker (HAL1843) in the ryanodine receptor gene for marker assisted selection [4], a burst of research looking for variation in DNA sequence took place in order to explain useful variation in economic traits. The availability of many genetic markers enabled the search for quantitative trait loci (QTL) using various breeding populations [5, 6] or by candidate gene approaches [7]. Typically, QTL studies made use of the higher variability of microsatellite markers and the generation of their maps. Candidate gene studies resulted in the identification of SNPs or indels. One of the key aspects for utility in marker assisted selection was the identification of consistent effects across different breeding populations or lines [8-10]. This increased confidence in the associations and simplified application. This across population marker relationship was essentially searching for markers that are in LD with causative mutations [11]. This desire for LD across populations meant that true associations within an individual line were often not used (type-2 error), but this was made up for by their more widespread utility. As the identification of large numbers of SNPs became easier then it was possible to explore the extent of LD in livestock, in order to determine the density required for different applications especially genome wide association studies. The availability of dense SNP panels (>50,000 SNPs per species) enables many different marker studies. Applications in the pig, include: inference on population history, structure and dynamics, estimation of effective population size, QTL mapping strategies, and whole genome association studies and genomic selection [12,13]. Therefore, knowledge of genomic diversity within and between populations is becoming more and more important in current livestock research. 

The development of high throughput genomic techniques provides us with an opportunity to assess genome wide diversity and its structure. Since the earlier draft sequence published for human (2001) and cattle (2009), an increasing number of studies have aimed at studying genomic diversity and LD in human and cattle [12-16]. For example, panels of more than 50,000 SNPs are available for cattle, pigs, sheep, chickens and horses and the latest panels for cattle contain more than 800,000 SNPs. The pig is now catching up these other species. To date most studies are based on a low marker density or a limited genomic region, which limits the utility of the results [1, 17-22]. The development of the Porcine 60K SNP panel helps to investigate genome wide diversity with a higher resolution [23]. Compared with other livestock, porcine populations are heavily structured and arranged in breeds for economic interest and environments. Comparative genomic diversity enables us to explore the degree of genomic variation and LD among pig and other species. This also helps to detect genomic regions that have been subject to selective sweeps in different pig populations. 

The focus of this review is on pig, however, we will compare the pig with results from human as well as other livestock species. We begin by discussing pig genomic diversity within sites and then addressed the genomic diversity among sites described by LD. In each case, we briefly compare pig genomic diversity with human and other livestock. The mechanisms for maintaining genomic diversity in pig are briefly discussed, including the factors of migration, introgression, selection and drift. A comprehensive review on population genomic diversity from the perspective of evolutionary theories is detailed in this volume by Hu *et al.* [24]. 

## 2. PIG GENOMIC DIVERSITY WITHIN SITES

### 2.1 Genetic Diversity within Populations

Pig genomic diversity within populations is quite variable. Observed heterozygosity ranged from 0.35 to 0.60, with an average ~ 0.5 across 17 autosomal chromosomes in 11 European pig breeds [25]. These values were similar to those in Chinese populations, ranging from 0.429 to 0.677 [19-20]. The expected heterozygosity was much higher than the observed heterozygosity in many reports. The average expected heterozygosity ranged from 0.53 to 0.80 among 13 populations from both Asia and Europe, including domestic and wild boars [20]. Chinese population had much higher diversity, ranging from 0.700 to 0.876 from 18 Chinese pig breeds [26]. If we consider a worldwide scale, pig populations had quite variable heterozygosities on different chromosomal regions. For example, the mean heterozygosity varied from 0.56 to 0.68 on chromosome 4 (SSC4) and from 0.65 to 0.80 on chromosome 7 (SSC7) in different populations [21]. This may reflect the relatively long time of breeding and selection for the pig.

A large number of SNPs are currently available for most species, such as human [27], cattle [15] and pig [23]. Recently, more than 372,000 SNPs were identified in swine, among which 45,510 SNPs were mapped to specific chromosomes in porcine genome build 7, including 21 SNPs on chromosome Y [23]. This number has now grown to more than 23 million (M. Groenen personal communication) with around 9M shared by both European and Chinese breeds. The number of SNPs varies largely on different chromosomes. With the exception of chromosome Y, most SNPs were identified on chromosome 1, followed by chromosomes 14, 4, and 7. Chromosome 12 had the least number of SNPs. This distribution partly reflects the cytogenetic map of the pig – chromosome 1 is the largest metacentric chromosome and 12 the smallest. The ratio of transitions to transversions was 2:1 [28-30], similar to that observed in human [31].

These SNPs were used to construct a panel using the Illumina platform, 64,232 SNPs were decoded and selected [23]. This panel shows quite a variable marker distribution among chromosomes and between genome builds as well as a proportion of unmapped SNPs, reflecting the incomplete state of knowledge of the pig genome to date. The average distance between SNPs on porcine genome build 7 was 30-40 kb, except for the X chromosome that had the larger distance (59.2 kb). This is close to the target required for efficient genome wide association studies according to estimates of LD. However, the largest distance between SNPs was around 450 kb on chromosomes 13 and 15, and the smallest gap was 161.2 kb on chromosome 18. The largest distances between SNPs in build 8 were generally higher than those in build 7. The number of intervals with a gap size larger than 250 kb was 115 and 207 in builds 7 and 8, respectively. In genome build 7, most gaps were on chromosomes 14 and X. Some chromosomes (2, 3, 6, 10, 12, 16 and 18) did not have any large gaps between the SNPs. In build 8, an increasing number of large gaps were observed for all chromosomes except for SSC14 where unmapped SNPs in build 7 were added in build 8, filling the observed large gaps [23]. These differences reflect the maturation of the genome sequence with different builds correcting some and introducing other errors. The latest build of the swine genome is build 10 which will be used for preparation of the sequence publication [32].

### 2.2. Genetic Diversity Among Populations

Since pig breeds from Asian and European populations were domesticated from different ancestors, they are expected to have different genetic diversities. One suggestion is that Asian wild boars had higher genetic diversity compared to European wild boars [2]. The nucleotide diversity in terms of Watterson’s method (θ) was 30% larger in Asian than in European populations [22]. Asian populations also have higher average heterozygosity than European populations. The observed and expected heterozygosities for worldwide breeds are summarized in Table **1** [19-20, 25-26]. It can be viewed that the average observed heterozygosity in Asian breeds was 0.566, ranging from 0.332 to 0.702, which in European breeds was around 0.542, at a range of 0.35 - 0.65. Furthermore, the average expected heterozygosity was also higher for Asian breeds (0.752) than for European breeds (0.570). Compared to domestic breeds, wild boars have a much higher observed heterozygosity, with an average of 0.628 and the range of 0.55 - 0.68. 

The genetic distance among populations is very varied reflecting the differences in domestication model and history in the two regions. We have summarized the *Nei’s* genetic distances between populations in Table **2** [19, 25, 26]. The average genetic distance in Asian populations was slightly higher than that in European populations. In Asian breeds, the average *Nei’s* genetic distance was 0.531, with a range from 0.194 to 1.188. In European populations, the average genetic distance was 0.495, ranging from 0.163 to 1.122. Higher average genetic distance was observed between European and Asian clades (1.434), which was nearly three times larger than those within populations. The smallest value was even up to 1.119 between Duroc and Tibet pigs [26]. However, Kim *et al*. [1] reported a smaller value (~ 0.017) between Asian and European pigs based on mtDNA D-loop sequences using the maximum-likelihood method, which was also much larger than that within Asian and European populations (~ 0.004).

Most genetic variation occurred within populations rather than between breeds from Asia and Europe. A summary of *F_ST_* from the literature [19, 25, 26] is provided in Table **2**. The average *F_ST_* value (0.257) between Asian and European populations was much higher than those within populations. When compared within the two different regions, the overall genetic differentiation for Asian populations was ~ 0.227, ranging from 0.182 to 0.294, much higher than that for European (0.134). The *F_ST_* value between domestic and wild boars for Asian (0.29) was also higher than that for European (0.194) [19, 25, 33]. A recent study using microsatellite markers also confirmed that the largest pairwise *F_ST_* was between Asian and European breeds (0.410, between Meishan and Hampshire), and the smallest was observed within European breeds (0.021, between Duroc and Yorkshire) [20]. 

All Asian and European pigs were closely related in terms of their maternal lineages, but they were different from each other. In wild boars, the average percentage sequence divergences calculated by mtDNA were 0.2370 within Chinese population, 0.3718 within European, and 0.5603 between Chinese and European. In domestic populations, the pairwise nucleotide sequence divergence was much lower (0.0056) between Chinese and European breeds [34]. Asian domestic populations had much larger variability than European breeds. Analysis based on mtDNA markers in 1536 samples (45 European and 21 Chinese breeds) indicated that the average frequency of mtDNA haplotypes in Asian was 29% of European breeds, but varied from 0 to 100% within individuals. A total of 28 Asian haplotypes were found in Chinese pigs, but only 6 Asian haplotypes were shared between European and Chinese populations [35]. These differences in genetic diversity are consistent with a strikingly different population history of humans and domestic animals. One separate study also revealed that the frequency of Asian haplotypes was low or absent in Duroc and Hampshire lines. The Landrace lines were less affected by Asian introgression than Large White lines. The haplotype in Pietrain which was originally developed in Belgium was completely absent in German and some commercial breeding lines, but showed a very high frequency of Asian haplotypes in some French lines [35]. It is worthwhile to note that Chinese breeds attributed high genetic diversity, with a higher level of haplotype diversity and smaller haplotype blocks than both wild boars and European breeds, but shared high levels of frequent haplotypes with Large White, Landrace, and Duroc. They also had a lower percentage of SNPs with the minor allele frequency (MAF) < 0.05. For instance, Meishan, a famous prolific Chinese breed, shared haplotypes that occurred at high frequency in European breeds [17]. 

### 2.3. A Comparison with Human and Other Livestock

A comparison of pig genomic diversity with human and other livestock is summarized in Table **3**. It shows that heterozygosity in pig was smaller than that in human and other livestock, but more flexible. Genetic variation within populations and genetic distance were much larger in pig than in human and other livestock. 

The average expected heterozygosity in human ranged from 0.7 to 0.9 [36], which was higher than the values observed in pig (~ 0.5). Other primates had lower heterozygosity than those in pig, such as 0.38 observed in gorilla [37] and 0.32 observed between Western and Eastern chimpanzee [38]. The genetic diversity between populations (*F_ST_*) was lower in human than in pig (0.021 - 0.410, Table **3**). In human, *F_ST_* was 0.05 - 0.13 for autosomal SNPs [39-43], ~ 0.11for autosomal copy number variations (CNVs) in a small set of populations, and 0.09 - 0.10 for *Alu* insertions polymorphism [43, 44]. Biswas *et al*. [14] indicated that 85% - 95% of human genetic variation was attributable to differences among individuals (higher heterozygosity), and that 5% - 15% was due to the differences between populations (lower *F_ST_*).

The genetic diversity was more extensive in goat than in pig (Table **3**). Recent studies [45, 46] using more than five southern Indian goat breeds based on microsatellite markers showed that the genetic diversity within breeds (*F_IS_*) ranged from 0.03 to 0.32. The observed and expected heterozygosities were at ranges of 0.42 - 0.67and 0.61 - 0.73, respectively. Among different goat breeds, *Nei’s* genetic distance differed from 0.067 to 0.830. The genetic diversity between breeds (*F_ST_*) ranged from 0.012 to 0.200. The overall proportion of genetic differentiation among breeds was around 0.14, such as 0.02 in Guadarrama goat [47], 0.06 and 0.17 in Egyptian and Italian goat breeds [48], 0.13 in north-western goat breeds of India [46], and 0.15 in indigenous goats of Sub-Saharan Africa [49]. Compared to pig, the average proportion of genetic diversity within goat breeds is much larger, and among breeds is much lower.

The average heterozygosity in cattle is slightly higher than that in pig (see Table **3**). The observed and expected heterozygosities in cattle were at the ranges of 0.47 - 0.74 and 0.45 - 0.78, respectively. While the genetic diversities both within (*F_IS_*) and between breeds (*F_ST_*) seem to be a slightly lower in cattle than in pig. *Nei’s *genetic distance was much larger in pig (0.163 - 1.794) than in cattle (0.015 - 0.382). Compared to pig, cattle genetic diversity has been more widely studied. Genetic diversity in Chinese breeds was higher than that in European breeds. This is similar to that found for the pig. In Chinese cattle breeds, the mean heterozygosity was at a range of 0.69 - 0.76, and Nei`s genetic distance ranged from 0.025 to 0.352 [50]. In European breeds, the observed and expected heterozygosities were 0.49 - 0.72 and 0.45 - 0.71, respectively. The genetic distance was at a range of 0.029 - 0.309 [51, 52]. In Brazilian breeds, the average observed and expected heterozygosity were much higher, ranging from 0.6316 to 0.7409 and 0.7151 to 0.7839, respectively. Genetic diversity within (*F_IS_*) and between (*F_ST_*) populations were slightly lower. Nei’s distance was at a range of 0.084 - 0.382 [53]. Pakistan cattle breeds displayed a moderate observed (0.47 - 0.51) and expected heterozygosity (0.63 - 0.67) [54]. 

## 3. PIG GENOMIC DIVERSITY AMONG SITES

### 3.1. LD within Populations

Within pig populations, a wide range of LD was observed on different chromosomes. With 29 and 5 microsatellite markers located on SSC15 and SSC2 in different lines, respectively, LD in terms of *r^2^* was higher for SSC2 (0.35 - 0.48) than for SSC15 (0.15 - 0.19) [55]. By investigating LD on SSC4 and SSC7 in five domestic pig populations, the mean *D´* was higher on SSC7 than on SSC4, and LD decreased faster with the marker distance on SSC4 than on SSC7 [21]. Using the Porcine 60K SNP Beadchip, **c**hromosomes 10 and 12 had the lowest average LD, chromosomes 1, 13, and 14 had the highest value [56]. Using the 60K panel, at the distance of 30 kb interval, the average LD on different autosomal chromosomes varied from 0.39 to 0.55 (*r^2^*). When considering longer SNP pair distance (3 Mb), differences in LD between different chromosomes were even larger up to 0.2 (for *r^2^* value) [56]. It indicated that LD distance in pig could extend to 3 Mb even with a higher value of *r^2^*. The significant LD variation among different regions was mainly due to the strong artificial selection on economic traits.

*D´* and *r^2^* are widely used to evaluate inter-site associations in livestock. *D´* strongly depends on allele frequency, and it decreases as the MAF increases. *r^2^* is more influenced by linkage distance. By using ~ 4,500 autosomal SNPs in more than 6,000 pigs from six commercial lines [18], the highest average *D´* was observed in case of MAF as 0.067. The *r^2^* values indicated that the largest LD was in the most tightly linked group. As the linkage distance increased from 0.1 to 40 cM, the average *r^2 ^*reduced from 0.371 to 0.008. The observed average *r^2^* began to rapidly decrease when the linkage distance approached to 3 cM. Amaral *et al*. [17] used high density SNP markers across 18 chromosomes in more than 20 pig breeds to evaluate pig LD. They suggested that LD extended to 1 - 3 cM in these pig populations, with *r^2^* being 0.3 as a threshold. Marker density and sampling size are the main factors that affect the accuracy of LD evaluation, which further influences the design of whole genome wide association studies. Nsengimana *et al*. [21] suggested that a marker density of 5 - 10 cM according to *D´* value was feasible in commercial pig populations for genome-wise association. Du *et al*. [18] recommended a considerably higher density of 0.1 to 1 cM for an initial whole genome scan. After a comparison among the distances from 40 - 60 kb [57] up to 400 kb [17] with commercial pig breeds, Ramos *et al*. [23] predicted that a density of 5 - 10 markers per cM was needed to conduct whole genome association studies in European breeds. However, a higher density is required for Asian breeds (see below).

### 3.2. LD AMONG POPULATIONS

The genome wide inter-site associations in terms of LD differ among different populations. Modern breeding programs increased the extent of LD and caused significant differences between European and Chinese pig breeds. LD decayed more rapidly in Chinese breeds than in European breeds, indicating that the extent of LD was smaller in Chinese breeds than in European breeds. In European breeds, LD extended up to 2 cM, and large haploblocks could be up to 400 kb; whereas in Chinese breeds, the extent of LD was smaller (0.05 cM) and generally did not exceed 10 kb [17, 58]. Chinese breeds had smaller LDs than European counterparts [17]. LD in Meishan breeds was larger than other Chinese breeds, but still smaller than that in most European breeds. When the whole genome was considered, the SNP spacing for European pig breeds was 0.1 cM, and 30,000 SNPs per individual were informative (with a MAF < 0.05 and *r^2^* = 0.3). However, for Chinese breeds with a similar sample size, the SNP spacing was 0.005 cM, and 500,000 SNPs per individual would be required [17]. This large difference in LD between European and Chinese breeds can be explained by the different ancestral stocks and modern breeding systems that produced smaller effective population sizes in Europe. However, both Chinese and European populations were not significantly different from wild boars in LD. The European wild boars showed an intermediate LD between Chinese and European domestic breeds [17]. 

Contrary to Chinese breeds, European breeds showed significant differences in LD [17]. By using the Porcine 60K SNP BeadChip [56], at the SNP distance interval of 5 Mb in Finnish breeding populations, Landrace displayed lower LD than Yorkshire. In Landrace, the percentages of adjacent SNP pairs with *r^2^* > 0.3 and *r^2 ^*> 0.2 were 49% and 57%, respectively; while the corresponding percentages for Yorkshire were 52% and 60%. At the average useful *r^2^* > 0.2, the SNPs density in Landrace and Yorkshire extended to 1.0 and 1.5 Mb, respectively, which resulted in a smaller effective population size (*N_e_*) for Yorkshire (55) than for Landrace (80). Similar *N_e_* were also obtained in American Landrace (74) and Berkshire (77), but higher in Hampshire (109) and Duroc (113) [59]. This, to some extent, reflected the different LDs in various populations. The variation of *D´* was also highly significant in Yorkshire, Large White and Landrace populations. The lowest *D´* was in Large White breeds, and the highest was in Duroc breeds [21]. 

### 3.3. Comparison of Pig LD with Human and other Livestock

A comparison of LD among pig, human, and other livestock is summarized in Table **4**. It shows that, at the same *r^2 ^*threshold, LD was significantly larger in pig than in human, but much smaller than in sheep. Compared with cattle, LD was much larger in pig at threshold of *r^2 ^*= 0.2 but similar to cattle at threshold of *r^2 ^*= 0.1. 

#### 3.3.1. Human

Due to a smaller effective population size and the stronger selection that has occurred in livestock, LD was significantly greater in pig (1 - 3 cM) than in human. The average LD in human was up to ~ 30 kb with a high variability, depending on the different populations and marker density used. Computer simulations and empirical data suggested that human LD extended only 3 - 5 kb for common disease SNPs, meaning that approximately 500,000 SNPs at least was needed for whole-genome studies [60, 61]. Later studies revealed that LD could extend to a distance greater than 100 kb in some cases [62, 63]. Among different populations, it revealed that LD in northern European populations was at a range of 10 - 30 kb, while LD in northern African populations was much lower [64]. Reich *et al*. [65] analyzed various extents of LD in different chromosomes based on common alleles and found levels of LD extending up to 40 - 160 kb in different regions. The United States population of north-European descent displayed LD extending 60 kb, which was greater than that in a Nigerian population. 

The LD information is very useful for estimating *N_e_* in different populations (see above for examples in pigs). For the SNP-pair interval less than 100 kb across all of the genome, the* N_e_* estimate was ~3100 for western European, Japanese and Chinese, and ~7500 for the Yoruba population [66]. This result supports the out-of-Africa theory of ancestral human population expansion and concurrent bottlenecks. Compared with human, pig has a significantly larger extent of LD (1 - 3 cM) and results in a smaller *N_e_* and fewer informative markers required for genome wide studies, at least for the European pig populations.

#### 3.3.2. Cattle

Generally, cattle have larger LD than human, but smaller LD than pig. LD in cattle was at a range of 40 - 100 kb at the threshold of *r^2^* > 0.2 [12, 15, 67]. de Roos *et al*. [12] compared 2430 animals from 5 breeds genotyped for 3072 SNP markers crossing 30 chromosomes. The average *r^2^* was 0.14 at marker distance of 100 kb. It indicated that at least 50,000 SNP markers were required for whole genomic selection. McKay *et al*. [13] reported the similar extensive LD by approximately 2670 genome markers within eight cattle breeds. They displayed that the average *r^2^* was 0.15 - 0.2 at a physical distance of 100 kb SNP loci apart, indicating that 30,000 - 50,000 markers were needed to conduct whole genome association studies. When microsatellite markers were used with four cattle breeds to estimate LD [16], at a marker-pair distance less than 5 cM, the average *r^2^* value across the populations was 0.16 at a range of 0.11 - 0.22. For the distance greater than 50 cM, the average *r^2^* declined to 0.07. Compared with cattle, pig LD was much stronger, especially for the useful *r^2^* > 0.2.

Extensive LD in cattle varied a little among different populations. In Holstein-Friesian cattle, by analyzing 1,566,890 syntenic and 365,400 non-syntenic SNP pairs that cover all autosomes [68], the significantly useful LD extended to 40 kb for *r^2^* and 8.2 Mb for *D´*. It indicated that at least 75,000 SNPs and a sample size of 75 or 400 would be required for whole genome association study. Qanbari *et al*. [15] used Illumina Bovine 50K SNP BeadChip to analyse LD structure in German Holstein-Friesian cattle. A mean value of 0.21 (*r^2^*) was observed for SNPs less than 100 kb apart. For American Holstein cattle, *r^2^* was much larger (0.59) at the same marker distance (100 kb) [69]. Kim & Kirkpatrick [70] also revealed strong LD (*r^2^* > 0.8) in genomic regions with marker distance less than 50 kb. LD for SNP pair intervals 100 kb apart (*r^2^* = 0.14) was similar to that in Holstein-Friesian cattle. Compared with pig, LD among populations varied less in cattle. This is in agreement with the common notion that a larger proportion of genetic variation exists within than between populations in most organisms. 

#### 3.3.3. Sheep

Compared with pig, LD in sheep seems much more extensive and flexible. High LD extending to 10 - 60 cM was observed in two domestic sheep breeds evaluated by microsatellite markers [71]. Large frequency of significant LD (*D`*) was observed for syntenic marker-pairs with the interval less than 60 cM. By using 490 markers in Soay sheep [72], 22% marker pairs had *D´* > 0.2 as for marker-pair distance < 10 cM. Meadows *et al*. [73] used 28 microsatellites in 555 animals from five sheep populations and obtained a flexible LD among different populations. Small LD ranging from 0 to 5 cM was obtained in five populations. In White Faced Suffolk, Poll Dorset and Macarthur Merino populations, average LD extended up to 20 cM in non-syntenic markers. A strong LD was observed at a marker-pair interval of 30 cM in several populations. The *D´* value between non-syntenic marker pairs ranged from 0.266 (Poll Dorset) to 0.322 (Merino × Border Leicester). *r^2 ^*decayed faster within Merino and Merino × Border Leicester. LD varied a lot among populations in sheep, similar to the results in pig.

## 4. MECHANISMS FOR MAINTAINING PIG GENOMIC DIVERSITY

### 4.1. Migration and Introgression

A significant differentiation in genetic diversity and LD exists between Asian and European pig populations. One reasonable explanation is that their domestications originated from different ancestors. However, they shared some important haplotypes with each other, which might come from extensive migration between populations and incomplete lineage sorting. Historic and demographic events revealed that Near Eastern pigs were definitely introduced into Europe during the 18 – 19^th^ centuries. When European wild boars were domesticated, they rapidly became the predominant lineage within European domestic swine [2]. So the nucleotide diversity in Europe had been heavily influenced by the migration from Asia [74]. There was also a widespread haplotype shared between breeds, but a significant genetic distance existed between European and Asian breeds [1, 17, 34, 35]. Even in a very low initial* N_e_*, subsequent migration can lead to a high level of polymorphism. While maternal introgression from European domestic pig has no or very little impact on Chinese breeds. That is why some European haplotypes were detected in some local breeds, but no European mtDNA haplotypes were detected [35].

### 4.2. Selection

Efficient artificial selection has a dramatic influence on livestock genome diversity and linkage disequilibrium. This effect depends on the direction, intensity, duration and consistency of selection over time. For a long time, in pigs selection focused on growth and fatness (representing demand for cheap lean meat). The availability of dense marker panels provides the opportunity to consider LD between regions and to search for signatures of selection as a means to identify causative mutations or other useful markers as has been done in several species. 

For example, two of the first QTL to be identified in the pig were on chromosomes 4 and 7. These include QTL for growth and fatness traits. However, despite significant efforts the genetic variation underlying these QTL has not been elucidated. The extent of LD for SSC7 was significantly larger compared to SSC4 [21]. Further analysis of LD in these regions found that Duroc and Landrace were under the strong selection pressure for both SSC4 and 7 while the selection effect for Large White and Yorkshire was only on SSC7. Interestingly, a significant difference in LD between SSC18 and SSC3 was also obtained (SSC18 > SSC3) that seemed to correlate with the numbers of candidate genes under selection, as several QTLs were mapped on SSC18 [17]. More recently Ojeda and colleagues have studied a number of gene regions under the SSC4 fatness QTL (FAT1) [22]. Although, they found significantly higher diversity for the region of the gene FABP4, they were unable to find any clear sign of a selective sweep in any of the breeds tested. This study did provide insight into the exchange of germplasm between China and Europe, and the authors concluded that an important part of variability within European breeds is due to introgression from Asian pigs. In this case migration is countering the loss of diversity caused by bottlenecks and artificial selection, with this mixing making it potentially harder to identify selective sweeps in pigs. 

Modern breeding practices and best linear unbiased prediction (BLUP) selection starting in the last century led to a rapid increase of genetic gain and large LD in pig breeds. This can cause inbreeding that increases LD but reduces genetic diversity. Taking Meishan for example, in order to increase the population size and reproductive performance, an inbred line of Meishan was developed in the late 1980s in a selection scheme. This may explain the lower genetic variability observed within Meishan [19] and that the level of LD was higher [17] compared to those in other Chinese populations.

Selection reduces genetic diversity on the next generation but enhances LD. Selection on one locus or multiple loci, will increase LD between the neighbouring loci and the selected locus. As a result of intensive artificial selection, livestock have a larger LD than human populations. In addition, selection can cause LD between unlinked loci that contribute to phenotypes undergoing selection [18]. This could also affect the structure of genomic diversity in livestock.

### 4.3. Drift

Domestication in livestock essentially results in two processes: the decline in *N_e_* (bottleneck effects) and directional artificial selection. The reduction in* N_e_* can lead to lower genetic diversity. LD initially generated by genetic drift gradually reduces with time. The genetic drift effects are substantial in historic pig breeds. The development of European and Chinese pig breeds has been different and the study of these differences can provide additional insights on diversity and these processes. It appears that the differences described to date are the result of differences in the number of ancestral stocks as well as the differences in modern breeding systems. Selection is likely to have been stronger in Europe and N America than in Asia. European breeds had smaller effective population sizes, resulting in stronger LD [17]. American Landrace and Berkshire had smaller *N_e_* than Hampshire and Duroc breeds, producing different LDs for the same marker-intervals [21, 59]. The joint effects of artificial selection and genetic drift produce different patterns of structured genomic diversity. 

## 5. CONCLUSION

In this review, we summarized genomic diversity within and between sites in pig (*Sus scrofa*) and compared them with human and other livestock. It is concluded that genetic diversity is smaller in pig than in human and other livestock. A relatively larger genetic variation within populations (*F_IS_*) exists in pig compared with cattle and goat. A majority of genetic variation in pig was distributed within rather than between populations, similar to human and other livestock. A large genetic distance exists between Asian and European populations. Asian breeds harbour much larger variability than European breeds. For the different genetic diversity structures, the inter-site associations described by LD were much stronger in pig (1 - 3 cM) than in human (~ 30 kb) and cattle (~ 100 kb), but less than in sheep (~ 10 cM). LD varied largely among different populations in pig and sheep, but a little in cattle. This review confirms that, strong breeding and selection programs have occurred in pig for a relatively long time, which resulted in the relatively lower heterozygosity and higher *F_IS_* and larger LD, compared with human and cattle. This also implies that a smaller effective population size and less informative markers would be needed for pig whole genome association studies (at least for European populations). To some extent, this difference might arise from the joint effects of migration, selection, and drift during the process of pig domestication. 

## Figures and Tables

**Table 1:** Summary of Observed and Expected Heterozygosity Estimates in European and Asian Breeds as well as Wild Boars Adapted from the Literature [19, 20, 25, 26]

	Observed Heterozygosity * (H_o_)*			Expected Heterozygosity* (H_e_)*		
	European	Asian	Wild	European	Asian	Wild
No of observations	17	28	5	17	28	5
Range	0.35 - 0.65	0.33 - 0.70	0.55 - 0.68	0.35 - 0.71	0.43 - 0.89	0.66 - 0.76
Average	0.542	0.566	0.628	0.570	0.752	0.708
S.D	0.078	0.087	0.050	0.095	0.132	0.039

**Table 2:** A Summary of Genetic Distance (*Nei*) and Genetic Variation (*F_ST_*) between Different Populations from the Published Literature [19,  25, 26]

	*Nei*			*F_ST_*		
	Within European	Within Asian	Between Asian and European	Within European	Within Asian	Between Asian and European
No of observations	58	174	52	10	3	15
Range	0.163 - 1.122	0.194 - 1.188	1.119 - 1.794	0.021 - 0.209	0.182 - 0.294	0.169 - 0.410
Average	0.495	0.531	1.434	0.134	0.227	0.257
S.D	0.189	0.188	0.166	0.055	0.059	0.080

**Table 3:** Comparison of Pig Genomic Diversity with Human and other Livestock Summarized from Published Results

Species	Within Populations			Between Populations		References
	*H_o_*	*H_e_*	*F_IS_*	*Nei*	*F_ST_*	
Pig	0.33 - 0.70	0.35 - 0.88	0.15 - 0.52	0.163 - 1.794	0.077 - 0.270	[19, 20, 25, 26]
Human	-	0.7 - 0.9	-	-	0.027 - 0.160	[14, 27, 75]
Cattle	0.47 - 0.74	0.45 - 0.78	0.05 - 0.26	0.015 - 0.382	0.028 - 0.216	[50-54]
Goat	0.42 - 0.67	0.61 - 0.73	0.03 - 0.32	0.067 - 0.830	0.012 - 0.200	[45, 46]

*H_o_* and *H_e_*: observed and expected heterozygosity, respectively. *F_IS_* and *F_ST_*: fixation indices of genetic diversity within and between populations. *Nei*: genetic distances between populations estimated by *Nei* standard method.

**Table 4:** Comparison of Pig LD Ranges with Human and other Livestock Summarized from Published Results

Species	*r^2^* range		References
	> 0.2	> 0.1	
Pig	< 3 Mb (cM)	< 5 Mb (cM)	[17, 18, 56]
Human	< 30 kb	< 100 kb	[64, 66]
Cattle	< 100 kb	< 5 cM	[12, 13, 15, 67, 68, 70]
Sheep	< 10 cM	< 30 cM	[73]

*r^2^*: LD estimated by *r^2^* value.

## References

[1] Kim KI, Lee JH, Li K, Zhang YP, Lee SS, Gongora J, Moran C (2002). Phylogenetic relationships of Asian and European pig breeds determined by mitochondrial DNA D-loop sequence polymorphism. Anim. Genet.

[2] Larson G, Dobney K, Albarella U, Fang M, Matisoo-Smith E, Robins J, Lowden S, Finlayson H, Brand T, Willerslev E, Rowley-Conwy P, Andersson L, Cooper A (2005). Worldwide phylogeography of wild boar reveals multiple centers of pig domestication. Science.

[3] Giuffra E, Kijas JMH, Amarger V, Carlborg, Jeon JT, Andersson L (2000). The origin of the domestic pig: independent domestication and subsequent introgression. Genetics.

[4] Plastow G, Siggens K, McLaren D (1994). genetic case study. Engineering biotechnologies for practical application to livestock. IEEE Potentials.

[5] Evans GJ, Giuffra E, Sanchez A, Kerje S, Davalos G, Vidal O, Illán S, Noguera JL, Varona L, Velander I, Southwood O I, de Koning DJ, Haley CS, Plastow GS, Andersson L (2003). Identification of quantitative trait loci for production traits in commercial pig populations. Genetics.

[6] Hu ZL, Park CA, Fritz ER, Reecy JM (2010). A Comprehensive Database Tool Building Bridges between Genotypes and Phenotypes. Invited Lecture with full paper published electronically on The 9th World Congress on Genetics Applied to Livestock Production. Leipzig, Germany, 2010.

[7] Wei W H, Skinner TM, Anderson JA, Southwood OI, Plastow G, Archibald AL, Haley CS (2010). Mapping QTL in the porcine MHC region affecting fatness and growth traits in a Meishan/Large White composite population. Anim. Gen.

[8] Rothschild MF, Newman S (2002). Intellectual Property Rights in Animal Breeding and Genetics. CABI Publishing.

[9] Rothschild M, Jacobson C, Vaske D, Tuggle C, Wang L, Short T, Eckardt G, Sasaki S, Vincent A, McLaren D, South-wood O, van der Steen H, Mileham A, Plastow G (1996). The estrogen receptor locus is associated with a major gene influencing litter size in pigs. Proc. Natl. Acad. Sci. USA.

[10] Short TH, Rothschild MF, Southwood OI, McLaren DG, de Vries A, van der Steen H, Eckardt GR, Tuggle CK, Helm J, Vaske DA, Mileham AJ, Plastow GS (1997). Effect of estrogen receptor locus on reproduction and production traits in four commercial lines of pigs. J. Anim. Sci.

[11] van der Steen HAM, Prall GFW, Plastow GS (2005). Application of genomics to the pork industry. J. Anim. Sci.

[12] de Roos APW, Hayes BJ, Spelman RJ, Godd ME (2008). Linkage disequilibrium and persistence of phase in Holstein Friesian, Jersey and Angus cattle. Genetics.

[13] McKay SD, Schnabel RD, Murdoch BM, Matukumalli LK, Aerts J, Coppieters W, Crews D, Neto ED, Gill C A, Gao C, Mannen H, Stothard P, Wang Z, van Tassell CP, Williams JL, Taylor JF, Moore SS (2007). Whole genome linkage disequilibrium maps in cattle. BMC Genet.

[14] Biswas S, Scheinfeldt LB, Akey JM (2009). Genome-wide Insights into the Patterns and Determinants of Fine-Scale Population Structure in Humans. Am. J. Hum. Genet.

[15] Qanbari S, Pimentel ECG, Tetens J, Thaller G, Lichtner P, Sharifi AR, Simianer H (2009). The pattern of linkage disequilibrium in German Holstein cattle. Anim. Genet.

[16] Upkin E, Straus K, Stein TR, Bagnato A, Schiavini F, Fontanesi L, Russo V, Medugorac I, Foerster M, Sölkner J, Dolezal M, Medrano JF, Friedmann A, Soller M (2009). Extensive long-range and nonsyntenic linkage disequilibrium in livestock populations: deconstruction of a conundrum. Genetics.

[17] Amaral AJ, Megens H, Crooijmans RPMA, Heuven HCM, Groenen MAM (2008). Linkage disequilibrium decay and haplotype block structure in the pig. Genetics.

[18] Du FX, Clutter AC, Lohuis MM (2007). Characterizing Linkage Disequilibrium in Pig Populations. Int. J. Biol. Sci.

[19] Fan B, Wang ZG, Li YJ, Zhao XL, Liu B, Zhao SH, Yu M, Li MH, Chen SL, Xiong TA, Li K (2002). Genetic variation analysis within and among Chinese indigenous swine populations using microsatellite markers. Anim. Genet.

[20] Luetkemeier ES, Sodhi M, Schook LB, Malhi RS (2010). Multiple Asian pig origins revealed through genomic analyses. Mol. Phylogenet. Evol.

[21] Nsengimana J, Baret P, Haley CS, Visscher PM (2004). Linkage disequilibrium in the domesticated pig. Genetics.

[22] Ojeda A, Ramos-Onsins SE, Marletta D, Huang LS, Folch J M, Pérez-Enciso M (2011). Evolutionary study of a potential selection target region in the pig. Heredity.

[23] Ramos AM, Crooijmans RPMA, Affara NA, Amaral AJ, Archibald AL, Beever JE, Bendixen C, Churcher C, Clark R, Dehais P, Hansen MS, Hedegaard J, Hu ZL, Kerstens HH, Law AS, Megens HJ, Milan D, Nonneman DJ, Rohrer GA, Rothschild MF, Smith TPL, Schnabel RD, Van Tassell CP, Taylor JF, Wiedmann RT, Schook LB, Groenen MAM (2009). Design of a high density SNP genotyping assay in the pig using SNPs identified and characterized by next generation sequencing technology. PLoS One.

[24] Hu XS, Yeh FC, Wang Z (2011). Structural genomics: correlation blocks, population structure, and genome architecture. Curr. Genomics.

[25] Laval G, Iannuccelli N, Legault C, Milan D, Groenen MAM, Giuffra E, Andersson L, Nissen PH, Jargensen CB, Beeckmann P, Geldermann H, Foulley JL, Chevalet C, Ollivier L (2000). Genetic diversity of eleven European pig breeds. Genet. Sel. Evol.

[26] Yang SL, Wang ZG, Liu B, Zhang GX, Zhao SH, Yu M, Fan B, Li MH, Xiong TA, Li K (2003). Genetic variation and relationships of eighteen Chinese indigenous pig breeds. Genet. Sel. Evol.

[27] (2010). The 1000 Genomes Project Consortium. A map of human genome variation from population-scale sequencing. Nature.

[28] Fahrenkrug SC, Freking BA, Smith TP, Rohrer GA, Keele JW (2002). Single nucleotide polymorphism (SNP) discovery in porcine expressed genes. Anim. Genet.

[29] Jungerius BJ, Rattink AP, Crooijmans RP, van der Poel JJ, van Oost BA, te Pas MF, Groenen MA (2003). Development of a single nucleotide polymorphism map of porcine chromosome 2. Anim. Genet.

[30] Kerstens HHD, Kollers S, Kommadath A, Rosario M, Dibbits B, Kinders SM, Crooijmans RP, Groene MAM (2009). Mining for Single Nucleotide Polymorphisms in Pig genome sequence data. BMC Genomics.

[31] Wang DG, Fan JB, Siao CJ, Berno A, Young P, Sapolsky R, Ghandour G, Perkins N, Winchester E, Spencer J, Kruglyak L, Stein L, Hsie L, Topaloglou T, Hubbell E, Robinson E, Mittmann M, Morris MS, Shen N, Kilburn D, Rioux J, Nusbaum C, Rozen S, Hudson TJ, Lipshutz R, Chee M, Lander ES (1998). Large-scale identification, mapping and genotyping of single nucleotide polymorphisms in the human genome. Science.

[32] Archibald AL, Bolund L, Churcher C, Fredholm M, Groenen MAM, Harlizius B, Lee KT, Milan D, Rogers J, Rothschild MF, Uenishi H, Wang J, Schook LB (2010). Pig genome sequence - analysis and publication strategy. BMC Genomics.

[33] Martinez AM, Delgado JV, Rodero A, Vega-Pla JL (2000). Genetic structure of the Iberian pig breed using microsatellites. Anim. Genet.

[34] Huang YF, Shi XW, Zhang Y P (1999). Mitochondrial Genetic Variation in Chinese Pigs and Wild Boars. Biochem. Genet.

[35] Fang M, Andersson L (2006). Mitochondrial diversity in European and Chinese pigs is consistent with population expansions that occurred prior to domestication. Proc. R. Soc. B.

[36] Carza JC, Slatkin M, Freimer NB (1995). Microsatellite allele frequencies in Humans and Chimpanzees, with implications for constraints on allele size. Mol. Biol. Evol.

[37] Thalmann O, Fischer A, Lankester F, Pääbo S, Vigilant L (2007). The complex evolutionary history of gorillas: insights from genomic data. Mol. Biol. Evol.

[38] (2005). The Chimpanzee Sequencing and Analysis Consortium. Initial sequence of the chimpanzee genome and comparison with the human genome. Nature.

[39] Auton A, Bryc K, Boyko A, Lohmueller K, Novembre J, Reynolds A, Indap A, Wright MH, Degenhardt J, Gutenkunst R, King KS, Nelson MR, Bustamante CD (2009). Global distribution of genomic diversity underscores rich complex history of continental human populations. Genome Res.

[40] Barreiro LB, Laval G, Quach H, Patin E, Quintana-Murci L (2008). Natural selection has driven population differentiation in modern humans. Nat. Genet.

[41] (2005). International Hap Map Consortium. A haplotype map of the human genome. Nature.

[42] Weir BS, Cardon LR, Anderson AD, Nielsen DM, Hill WG (2005). Measures of human population structure show heterogeneity among genomic regions. Genome Res.

[43] Xing J, Watkins WS, Witherspoon DJ, Zhang Y, Guthery SL, Thara R, Mowry BJ, Bulayeva K, Weiss RB, Jorde LB (2009). Fine-scaled human genetic structure revealed by SNP microarrays. Genome Res.

[44] Redon R, Ishikawa S, Fitch KR, Feuk L, Perry GH, Andrews TD, Fiegler H, Shapero MH, Carson AR, Chen W, Cho EK, Dallaire S, Freeman JL, González JR, Gratacòs M, Huang J, Kalaitzopoulos D, Komura D, MacDonald JR, Marshall CR, Mei R, Montgomery L, Nishimura K, Okamura K, Shen F, Somerville MJ, Tchinda J, Valsesia A, Woodwark C, Yang F, Zhang J, Zerjal T, Zhang J, Armengol L, Conrad DF, Estivill X, Tyler-Smith C, Carter NP, Aburatani H, Lee C, Jones KW, Scherer SW, Hurles ME (2007). Global variation in copy number in the human genome. Nature.

[45] Bruno-de-Sousa C, Martinez AM, Ginja C, Santos-Silva F, Carolino MI, Delgado JV, Gama LT (2010). Genetic diversity and population structure in Portuguese goat breeds. Livest. Sci.

[46] Dixit SP, Verma NK, Aggarwal RAK, Kumar S, Chander R, Vyas MK, Singh KP (2009). Genetic structure and differentiation of three Indian goat breeds. Asian-Aust. J. Anim. Sci.

[47] Serrano M, Calvo JH, Martinez M, Marcos-Carcavilla A, Cuevas J, Gonzallez C, Jurado JJ, Tejada PD (2009). Microsatellite based genetic diversity and population structure of the endangered Spanish Guadarrama goat breed. BMC Genet.

[48] Agha SH, Pilla F, Galal S, Shaat I, Andrea MD, Reale S, Abdelsalam AZA, Li MH (2008). Genetic diversity in Egyptian and Italian goat breeds measured with microsatellite polymorphism. J. Anim. Breed. Genet.

[49] Chenyambuga SW, Hanotte O, Hirbo J, Watts PC, Kemp SJ, Kifaro GC, Gwakisa PS, Petersen PH, Rege JEO (2004). Genetic characterization of indigenous goats of Sub-Saharan Africa using microsatellite DNA markers. Asian-Aust. J. Anim. Sci.

[50] Zhou GL, Jin HG, Zhu Q, Guo SL, Wu YH (2005). Genetic diversity analysis of five cattle breeds native to China using microsatellites. J. Genet.

[51] Canon J, Alexandrino P, Bessa I, Carleos C, Carretero Y, Dunner S, Ferran N, Garcia D, Jordana J, Laloe L, Pereira A, Sanchez A, Moazami-Goudarzi K (2001). Genetic diversity measures of local European beef cattle breeds for conservation purposes. Genet. Sel. Evol.

[52] Kantanen J, Olsaker I, Holm LE, Lien S, Vilkki J, Brusgaard K, Eythorsdottir E, Danell B, Adalsteinsson S (2000). Genetic diversity and population structure of 20 north European cattle breeds. J. Hered.

[53] Egito AA, Paiva SR, Albuquerque MSM, Mariante AS, Almeida LD, Castro SR, Grattapaglia D (2007). Microsatellite based genetic diversity and relationships among ten Creole and commercial cattle breeds raised in Brazil. BMC Genet.

[54] Rehman MS, Khan MS (2009). Genetic diversity of hariana and hissar cattle from Pakistan using microsatellite analysis. Pakistan Vet. J.

[55] Harmegnies N, Farnir F, Davin F, Buys N, Georges M, Coppieters W (2006). Measuring the extent of linkage disequilibrium in commercial pig populations. Anim. Genet.

[56] Uimari P, Tapio M (2010). Extent of linkage disequilibrium and effective population size in Finnish Landrace and Finnish Yorkshire pig breeds. J. Anim. Sci.

[57] Jungerius BJ, Gu J, Crooijmans RPMA, Poel JJ, Groenen MAM, van Oost BA, te Pas MF (2005). Estimation of the extent of linkage disequilibrium in seven regions of the porcine genome. Anim. Biotechnol.

[58] Megens HJ, Crooijmans RPMA, Heuven HCM, Groenen MAM (2008). Linkage disequilibrium decay and haplotype block structure in the pig. Genetics.

[59] Welsh CS, Stewart TS, Schwab C, Blackburn HD (2010). Pedigree analysis of 5 swine breeds in the United States and the implications for genetic conservation. J. Anim. Sci.

[60] Dunning AM, Durocher F, Healey CS, Teare MD, McBride SE, Carlomagno F, Xu CF, Dawson E, Rhodes S, Ueda S, Lai E, Luben RN, van Rensburg EJ, Mannermaa A, Kataja V, Rennart G, Dunham I, Purvis I, Easton D, Ponder BAJ (2000). The extent of linkage disequilibrium in four populations with distinct demographic histories. Am. J. Hum. Genet.

[61] Kruglyak L (1999). Prospects for whole-genome linkage disequilibrium mapping of common disease genes. Nat. Genet.

[62] Abecasis GR, Noguchi E, Heinzmann A, Traherne JA, Bhat-tacharyya S, Leaves NI, Anderson GG, Zhang Y, Lench NJ, Carey A, Cardon LR, Moffatt MF, Cookson WOC (2001). Extent and distribution of linkage disequilibrium in three genomic regions. Am. J. Hum. Genet.

[63] Taillon-Miller P, Bauer-Sardiña I, Saccone NL, Putzel J, Laitinen T, Cao A, Kere J, Pilia G, Rice JP, Kwok PY (2000). Juxtaposed regions of extensive and minimal linkage disequilibrium in human Xq25 and Xq28. Nat. Genet.

[64] Ardlie KG, Kruglyak L, Seielstad M (2002). Patterns of linkage disequilibrium in the human genome. Nat. Rev. Genet.

[65] Reich DE, Cargill M, Bolk S, Ireland J, Sabeti PC, Richter DJ, Lavery T, Kouyoumjian R, Farhadian SF, Ward R, Lander ES (2001). Linkage disequilibrium in the human genome. Nature.

[66] Tenesa A, Navarro P, Hayes BJ, Duffy DL, Clarke GM, Goddard ME, Visscher PM (2007). Recent human effective population size estimated from linkage disequilibrium. Genome Res.

[67] Flury C, Tapio M, Sonstegard T, Drögemüller C, Leeb T, Simianer H, Hanotte O, Rieder S (2010). Effective population size of an indigenous Swiss cattle breed estimated from linkage disequilibrium. J. Anim. Breed. Genet.

[68] Khatkar MS, Nicholas FW, Collins A Q, Zenger KR, Cavanagh JAL, Barris W, Schnabel RD, Taylor JF, Raadsma HW (2008). Extent of genome-wide linkage disequilibrium in Australian Holstein-Friesian cattle based on a high-density SNP panel. BMC Genomics.

[69] Sargolzaei M, Schenkel FS, Jansen GB, Schaeffer LR (2008). Extent of linkage disequlibrium in Holstein cattle in North America. J. Dairy Sci.

[70] Kim ES, Kirkpatrick BW (2009). Linkage disequilibrium in the North American Holstein population. Anim. Genet.

[71] McRae AF, McEwan JC, Dodds KG, Wilson T, Crawford AM, Slate J (2002). Linkage disequilibrium in domestic sheep. Genetics.

[72] McRae AF, Pemberton JM, Visscher PM (2005). Modeling linkage disequilibrium in natural populations: the example of the soay sheep population of St. Kilda, Scotland. Genetics.

[73] Meadows JRS, Chan EK, Kijas JW (2008). Linkage disequilibrium compared between five populations of domestic sheep. BMC Genet.

[74] Ramirez O, Ojeda A, Tomas A, Gallardo D, Huang LS, Folch JM, Clop A, Sánchez A, Badaoui B, Hanotte O, Gal-man-Omitogun O, Makuza SM, Soto H, Cadillo J, Kelly L, Cho IC, Yeghoyan S, Pérez-Enciso M, Amills M (2009). Integrating Y-chromosome, mitochondrial and autosomal data to analyse the origin of pig breeds. Mol. Biol. Evol.

[75] Xing J, Watkins WS, Shlien A, Walker E, Huff CD, Witherspoon DJ, Zhang Y, Simonson TS, Weiss RB, Schiffman JD, Malkin D, Woodward SR, Jorde LB (2010). Toward a more uniform sampling of human genetic diversity: A survey of worldwide populations by high-density genotyping. Genomics.

